# The Impact of Artificial Restoration of Alpine Grasslands in the Qilian Mountains on Vegetation, Soil Bacteria, and Soil Fungal Community Diversity

**DOI:** 10.3390/microorganisms12050854

**Published:** 2024-04-25

**Authors:** Xiaomei Yang, Qi Feng, Meng Zhu, Jutao Zhang, Linshan Yang, Ruolin Li

**Affiliations:** Key Laboratory of Ecohydrology of Inland River Basin, Alax Desert Eco-Hydrology Experimental Research Station, Qilian Mountains Eco-Environment Research Center in Gansu Province, Northwest Institute of Eco-Environment and Resources, Chinese Academy of Sciences, Lanzhou 730000, China; yangxiaomei21@nieer.ac.cn (X.Y.); zhumeng@lzb.ac.cn (M.Z.); jutzhang@lzb.ac.cn (J.Z.); yanglsh08@lzb.ac.cn (L.Y.); ruolinli@lzb.ac.cn (R.L.)

**Keywords:** alpine mining area grassland, artificial restoration, vegetation diversity, microbial diversity, Qilian Mountains

## Abstract

To understand how the soil microbial community structure responds to vegetation restoration in alpine mining areas, this study specifically examines the grassland ecosystem in the Qianmalong mining area of the Qilian Mountains after five years of artificial restoration. High-throughput sequencing methods were employed to analyze soil bacteria and fungi microbial characteristics in diverse grassland communities. Combined with modifications in vegetation diversity as well as soil physicochemical properties, the impact of vegetation restoration on soil microbiome diversity in this alpine mining area was investigated. The findings indicated that the dominant plants were *Cyperus rotundus*, *Carex* spp., and *Elymus nutans*. As the extent of the grassland’s restoration increased, the number of plant species, importance values, and plant community diversity showed an increasing trend. The plant functional groups were mainly dominated by Cyperaceae, followed by Poaceae. Plant height, density, plant cover, frequency, and aboveground biomass showed an increasing trend, and soil water content (SWC) increased. While soil pH and soil electrical conductivity (EC) exhibited a declining trend, available phosphorus (AP), total phosphorus (TP), total nitrogen (TN), nitrate nitrogen (NO_3_-N), soil organic carbon (SOC), and soil water content (SWC) showed an increasing trend. The dominant bacterial communities were Actinobacteriota, Proteobacteria, Acidobacteriota, Chloroflexi, Firmicutes, and Gemmatimonadota, while the dominant fungal communities were Ascomycota, Mortierellomycota, Basidiomycota, unclassified_k_Fungi, and Glomeromycota. Significant differences were detected within soil microbial community composition among different degrees of restoration grasslands, with bacteria generally dominating over fungi. SWC, TP, and TN were found to be the main soil physicochemical factors affecting the distribution of soil bacterial communities’ structure; however, SOC, TN, and NO_3_-N were the primary factors influencing the soil distribution of fungal communities. The results of this study indicate that different degrees of vegetation restoration in alpine mining areas can significantly affect soil bacterial and fungal communities, and the degree of restoration has varying effects on the soil bacteria and fungi community structure in alpine mining areas.

## 1. Introduction

The Qilian Mountains National Park serves as a crucial water conservation area and ecological barrier in Northwest China, playing a vital role in ecological preservation, cultural development, and economic advancement along the Hexi Corridor [1]. Large-scale mining activities began in the early 1980s, resulting in extensive environmental degradation due to excessive mining within the Qilian Mountains. Consequently, the alpine grassland ecosystem in the mining areas became highly sensitive and fragile, with severe damage to vegetation diversity and soil [2,3]. Remnants of mining activities have left the surrounding ecological environment compromised, with numerous slag heaps scattered throughout river channels and adjacent grasslands, leading to varying degrees of degradation in vegetation and soil quality [4,5]. In recent years, land reclamation and ecological restoration in mining areas have become focal points of both domestic and international concern. The execution of the Qilian Mountains ecological conservation and restoration project is of significant importance to enhancing the ecological environment along the Hexi Corridor and constructing an ecological security buffer zone in Western China [6,7].

Vegetation restoration is not only a prerequisite for ecosystem recovery but also an effective measure to improve the ecological system of mining-area grasslands [8,9]. Vegetation, as a producer in the grassland ecosystems of mining areas, plays a vital role in both the artificial and natural restoration of these ecosystems [10]. Following mining activities, the vegetation cover in mining areas is typically very low or nearly absent, leading to severe soil erosion and loss of soil, as well as significant damage to soil structure. This disruption in the energy flow and material cycling in mining-area grassland ecosystems creates a vicious cycle, disrupting the balance of the ecosystem [11]. In the restoration of vegetation in mining-area ecosystems, it is essential to consider the selection and combination of native species to enhance resistance and richness. Moreover, it is crucial to adhere to the principle of coordinated soil and vegetation restoration to address issues such as soil fertility and nutrient deficiencies. Increasing vegetation diversity can promote soil microbial diversity, and the increase in diversity can enhance the stability of mining area grassland ecosystems [12]. In the Qilian Mountain alpine mining area, natural restoration is primarily employed, supplemented by artificial ecological restoration. Measures such as covering the soil with vegetation and coordinating with the surrounding environment have initially achieved the goals of restoration and management [13].

Soil microbes are highly sensitive to changes in ecosystems, and their diversity is essential for maintaining the sustainable development of the environment, playing a crucial role in vegetation restoration and growth [14]. Alterations in soil microbiological composition are extremely sensitive to factors such as soil type, dominant species, soil pH, and geographical environment, indicating soil physicochemical properties and functions [15]. While both artificial and natural restoration aim to restore aboveground vegetation diversity, the process of vegetation restoration involves interactions between soil microbes and vegetation [16]. During the vegetation restoration process in mining areas, significant changes occur in the community structure, composition, and function of soil microorganisms. Different vegetation restoration methods and degrees of restoration result in varied changes in the soil, leading to differences in soil ecosystem dynamics [17]. Therefore, understanding the influence of alpine mining activities on the recovery of soil microorganism community diversity in grasslands and exploring the correlation between varying degrees of restoration and soil microbial diversity is of essential theoretical and practical significance for guiding vegetation restoration in alpine mining-area grassland ecosystems. This study examines grasslands at various stages of restoration in alpine mining regions, examining the changes in soil microbial communities in response to vegetation restoration and providing a theoretical basis for exploring vegetation restoration in alpine mining areas in the Qilian Mountains.

## 2. Materials and Methods

### 2.1. Study Region

The study area is situated within the eastern end of the Qilian Mountains Nature Reserve, in the southwestern part of the Tianzhu Tibetan Autonomous County, Gansu Province, China. Specifically, it encompasses the Qianmalong coal mining area, with a total area of 1.0235 km^2^. The Qianmalong coal mine commenced operations in 2014, with an annual production capacity of 90,000 t. Mining operations reached depths between 2620 and 2800 m before being officially closed in April 2017 due to policy reasons [18]. Over the years, the ecological environment in the area was severely affected by mineral resource exploitation, as inadequate protection measures were in place. Consequently, the grassland ecosystem in the mining area has experienced significant degradation. Since 2018, in the Qianmalong coal mine area, restoration has been primarily achieved through artificial means, and efforts have been made to restore the grassland ecosystem through a comprehensive management approach, primarily focusing on artificial restoration with limited natural regeneration. Key restoration measures include the leveling of coal gangue slag heaps, demolition of abandoned buildings, backfilling of abandoned mines, soil covering, and vegetation restoration. These restoration efforts have led to noticeable changes in vegetation coverage, greatly improving the ecological environment of the mining region.

**Figure 1:**
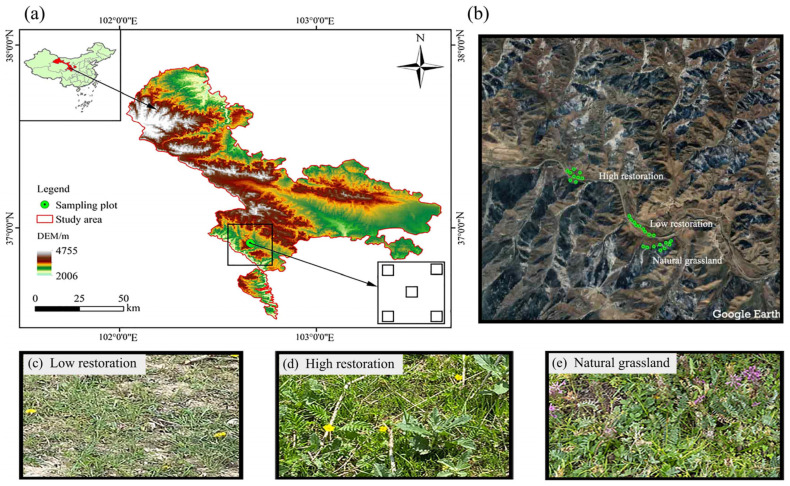
Location of the study area in the Chinese Qilian Mountains (**a**,**b**); LR, low restoration (**c**); HR, high restoration (**d**); and NG, natural grassland serving as the CK (**e**).

The climate of the study area is characterized by a high-altitude, semi-arid climate; the average temperature is 0.9 °C, and the average annual precipitation is 500–650 mm, with total evaporation averaging between 1000 and 1100 mm (Figure 1). Precipitation is concentrated between June and September, with the highest rainfall occurring in July and August. The primary water system is affiliated with the Jingshaxia River, a first-level tributary of the Datong River, which serves as a potential source for vegetation greening. The mining area is predominantly located in a mountainous region with complex terrain and significant elevation differences. Because of the combined effects of topography, climate, and human activity, soil types vary within the mining area. In the mountainous regions of the mine, meadow steppe soil predominates, followed by dark chernozem and black loam. In the piedmont plains of the mining area, fluvisols and vertisols are the primary soil types. Vegetation is predominantly composed of species such as *Cyperus rotundus*, *Carex* spp., and *Polygonum viviparum*.

### 2.2. Plotting and Sample Collection

Through interviews with local herdsmen, a literature review, and on-site investigation, grassland recovery progress at different levels of rehabilitation in the Qianmalong mining area was assessed. The restoration of succession sequences relies on China’s “Soil Erosion Classification and Grading Standard” (SL190-2007). Beginning in 2018, restoration activities, including debris removal and seed sowing, were initiated based on the vegetation cover of the natural recovery process. Restoration levels are classified based on extent of vegetation cover into high and low restoration. Grasslands with high restoration have a vegetation cover of over 70%, whereas those with low restoration have a vegetation cover greater than 20% but less than 40%. The restored alpine mining grasslands were categorized into three levels: low restoration (LR), high restoration (HR), and natural grassland (NG), with NG serving as the control check (CK) (Table 1). The control group consists of natural grassland primarily located in the steppe region, where the coverage exceeds 68%. The soil is classified as meadow steppe soil, which shares similar topographic and climatic conditions with the grasslands in the restoration area. To ensure the representativeness of the sampling sites, three sites were selected for each level of recovery and the control group, with five soil sampling points established within each site. This resulted in a total of 9 sites and 45 sampling points. Field vegetation surveys and sample collection were conducted in July 2022 (Figure 1). A quadrat sampling method was employed to survey plant communities. At each sampling point, three plots representing were randomly selected. Within each plot, three 1 × 1 m quadrats were established. Vegetation community characteristics, including species composition, richness, cover (estimated visually), abundance (number of individuals per species within the quadrat), and aboveground biomass, were recorded during the surveys [19]. We computed the species diversity of vegetation communities at different restoration levels based on their relative abundance. Diversity measures included the Simpson index, Shannon–Wiener index, Margalef index, dominance index, and evenness index.

Soil sampling was conducted simultaneously with the vegetation survey in our study area. Within each level of plant recovery, three large plots (200 × 200 m) were established. Within each large plot, three small plots (50 × 50 cm) were further designated. Soil samples were obtained from the 0–20 cm soil horizon using soil augers. A portion of the samples was promptly placed in dry ice and transported to the laboratory for the analysis of soil microbial community diversity. Other portions of fresh soil samples were placed into sterilized self-sealing bags for analysis of soil characteristics. Determination of SWC was conducted using the drying method. The soil pH, EC, SOC, TN, NO_3_-N, TP, and AP were analyzed using the following methods: electrode potential for pH, conductivity meter for EC, dichromate oxidation for SOC, element analyzer for TN, interval-flow analysis for NO_3_-N, alkaline solution–diffusion for TP, and molybdenum antimony anti-colorimetric methods for AP [20,21].

### 2.3. Sample Analysis

Vegetation species richness was determined using the quadrat method, counting the number of species in each quadrat. Different vegetation types were assessed for plant height, cover, and frequency. Vegetation samples from different vegetation types were clipped, weighed, and placed in envelopes for transportation to the laboratory. After being dried at 65 °C for 48 h, aboveground biomass was obtained. Relative cover, relative frequency, relative biomass, and relative abundance of different plants were calculated to determine their importance values within the plant community.

Soil microbial diversity was initially assessed by extracting genomic DNA from soil samples using the CTAB (Cetyltrimethylammonium bromide) method. Following purity and concentration determination, the DNA was diluted to a concentration of 1 ng·μL^−1^. Diluted soil genomic DNA was used as a prototype, the 16S rRNA gene of soil bacteria was amplified by PCR with specific primers 515F and 806R, with three replicates. The library construction utilized the TruSeq^®^ DNA PCR-Free Sample Preparation Kit (1 November 2023), and after quantification using qPCR, qualified libraries were subjected to sequencing using the NovaSeq6000 platform (1 November 2023). Soil fungi were amplified using ITS primers 1737F and 2043R via PCR and subsequently subjected to Illumina library (1 November 2023) preparation and sequencing [22].

### 2.4. Bioinformatics Analysis

The determination of different samples’ data was based on the amplification of PCR primer sequences. After concatenating reads from all samples, raw data were obtained and subjected to quality control and removal of chimeras to obtain valid data. The Uparse software (1 December 2023) was utilized to cluster all sample data into operational taxonomic units (OTUs) at a similarity throttle of 97%. Species annotation was carried out using the Mothur method in conjunction with the Silva software database (1 December 2023), with a threshold set at 0.8–1.0. The valid data sequences were aligned employing MUSCLE software (version 3.8.31), and the sequencing data were standardized simultaneously. Soil microbial alpha diversity indices and beta diversity indices were calculated using R3.6 software [23]. Non-metric multidimensional scaling (NMDS) was employed with OTU level to assess differences between soil bacterial and fungal communities across samples.

### 2.5. Data Analysis

The experimental data underwent processing and analysis with SPSS 20.0 (IBM) and Microsoft Excel 2020 software, with Sigmaplot 12.5 being used for graphical representation. General linear models were employed for repeated measurements of all data. The microbial abundance in grasslands at different recovery levels in the mining area was analyzed using one-way ANOVA single-factor variance analysis, and subsequent post hoc tests were conducted to identify significant variations. Principal component analysis (PCA) and related analyses were performed to pinpoint the primary environmental factors influencing variations in vegetation and soil bacterial and fungal communities in the alpine mining-area grassland. Redundancy analysis (RDA) was employed to assess the contribution of environmental factors for vegetation and soil to changes in soil bacterial and fungal communities in the restored grasslands of the alpine mining area. This was achieved using CANOCO 5.0, a software package for ordination and constrained analysis of correspondence. Spearman’s related analysis was used to explore the relationships between vegetation and soil properties, as well as phyla and classes of soil bacteria and fungi with a relative abundance exceeding 1%. Co-occurrence networks of soil bacteria and fungi were constructed through Spearman’s correlation analysis, facilitated by the *Networkx* module for Python version 3.9 and Gephi version 0.9. The analysis utilized the pheatmap package in R version 3.3.1 (pheatmap package) [24].

## 3. Results

### 3.1. Traits of Vegetation Communities within Alpine Grasslands of Mining Regions across Varying Restoration Levels

After 5 years of artificial vegetation succession, significant differences have been observed in the composition of plant communities within the elevated mining zone in the Qilian Mountains at different levels of recovery. There are notable differences in the dominant species among the three recovery levels (Table 2). The dominant species are primarily dominated by *Cyperus rotundus*, with variations in the proportion of perennial plants among grasslands at different recovery levels, accounting for 87.09%, 93.58%, and 91.50% in LR, HR, and NG, respectively. Specifically, the subdominant species in LR recovery grasslands were *Elymus nutans* + *Daucus carota*, while in HR recovery grasslands, they were *Artemisia smithii Mattf* + *Chenopodium glaucum*, and in NG, they were *Potentilla chinensis* + *Oxytropis ochrocephala*.

### 3.2. Effects of Vegetation Restoration in Alpine Mining Areas on Grassland Species Diversity

**Figure 2:**
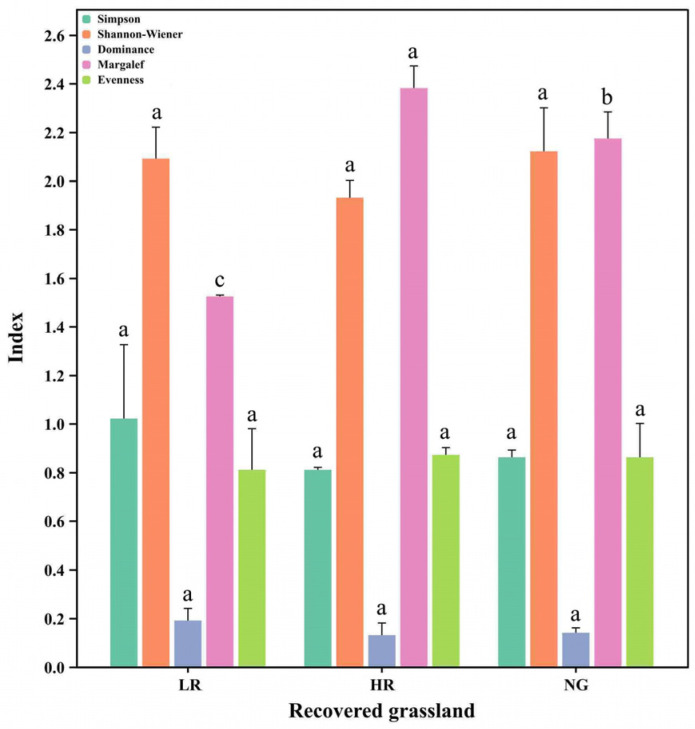
Variations in the diversity of grassland vegetation communities across various restoration stages. LR, low restoration; HR, high restoration; NG, natural grassland. Different letters on the back of the values between treatments indicate significant differences at the 0.05 level.

The impact of different recovery levels on vegetation species diversity in the high-altitude mining area exhibits inconsistent trends (Figure 2). Analysis of plant community diversity across different recovery levels reveals that Simpson’s diversity index shows no significant difference, with the highest value observed in LR at 0.87. Similarly, the Shannon–Wiener index demonstrates no significant differences, with the highest value observed in NG at 2.12. However, the dominance index (HR and LR) demonstrates substantial variations (*p* < 0.05), with HR having the lowest dominance index at 0.13. The species richness index also exhibits marked disparities (*p* < 0.05), with the maximum value recorded in HR at 2.73, indicating the best recovery level. The evenness index showed no notable disparities, where NG recorded the maximum at 0.88, with HR trailing closely behind at 0.84.

### 3.3. Effects of Vegetation Restoration on Soil Properties in Alpine Mining Areas

**Figure 3:**
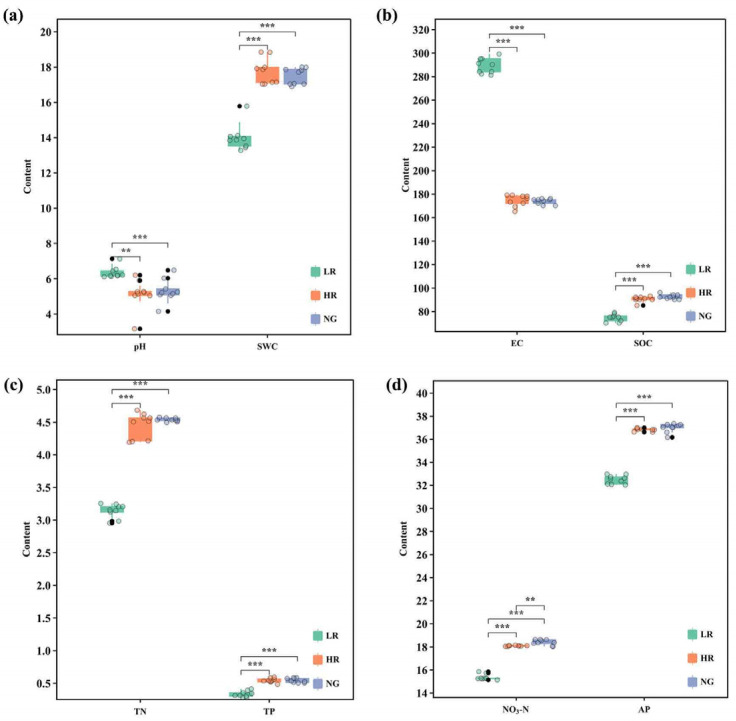
Changes in physicochemical properties of grassland soils in mining zones featuring various levels of restoration. LR, low restoration; HR, high restoration; NG, natural grassland. pH, soil pH; EC, soil electroconductibility; SWC, soil water content; SOC, soil organic carbon; TN, soil total nitrogen; AP, soil available phosphorus; TP, soil total phosphorus; NO_3_-N, soil nitrate nitrogen. (**a**) pH and SWC content; (**b**) EC and SOC content; (**c**)TN and TP content; (**d**) NO_3_-N and AP content, ** indicates extremely significant correlation (*p* < 0.01), *** indicates extremely significant correlation (*p* < 0.001), black circle indicates outliers, same as below.

The impact of different recovery levels on soil physical and chemical characteristics in the elevated mining zone showed significant differences (Figure 3). Analysis of soil properties across different recovery levels indicates that the soil pH in areas with higher recovery levels was significantly lower than that in areas with lower recovery levels (*p* < 0.05). Soil moisture content demonstrates a significant increasing trend with increasing recovery levels (*p* < 0.05), with values of 14.51%, 17.92%, and 17.87%, respectively (Figure 3a). Soil conductivity exhibits a significant decreasing trend with increasing recovery levels (*p* < 0.01), with values of 292.67 μS/cm, 173.33 μS/cm, and 173.67 μS/cm, respectively. Soil organic carbon increases with increasing recovery levels (Figure 3b). Soil total nitrogen exhibited a notable increasing pattern across increasing recovery levels (*p* < 0.05), with values of 3.12 g/kg, 4.47 g/kg, and 4.54 g/kg, respectively (Figure 3c). Soil total phosphorus increased with increasing recovery levels. Soil nitrate nitrogen and soil available phosphorus both showed significant increasing trends with increasing recovery levels (*p* < 0.05) (Figure 3d). The soil physicochemical properties of HR and NG tended to be consistent, indicating the best recovery effects. With maintenance and changes in recovery levels over time, soil properties can approach those of natural grasslands. Among the recovery methods within the elevated mining zone, the HR method yields the best results.

### 3.4. Impact of Vegetation Restoration on Soil Bacterial Communities in Alpine Mining Areas

#### 3.4.1. Structure of Soil Bacterial Populations in Grassland Ecosystems within Mining Zones at Varying Stages of Ecological Restoration

**Figure 4:**
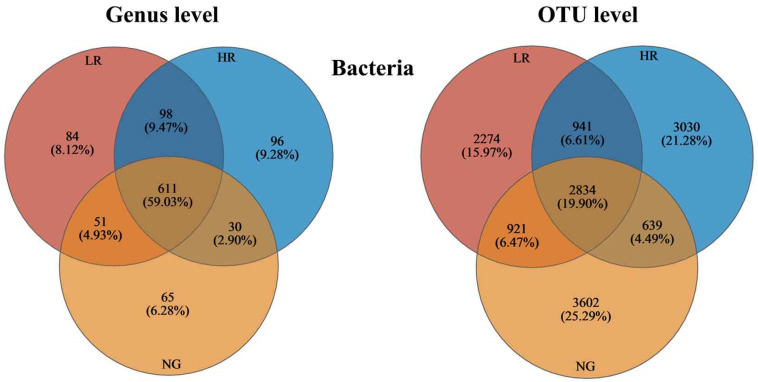
Numbers and proportions of common and unique genera and OTUs of soil bacteria in grasslands in mining zones featuring various levels of restoration.

In the grasslands of mining areas with different recovery levels (LR, HR, and NG), there were 611 genera shared across all recovery levels, accounting for 59.03% of the total genera, and 2834 shared OTUs, accounting for 19.90% of the total OTUs (Figure 4). Specifically, both the genera and OTUs of soil bacteria in HR sites were higher than those in LR sites (96 > 84 and 3030 > 2274, respectively). The number of soil bacteria genera in HR sites was higher than that in NG sites (96 > 65), while the number of soil bacteria OTUs in NG sites was higher than that in HR sites (3602 > 3030).

#### 3.4.2. Relative Abundance of Soil Bacterial Community Composition in Grasslands of Mining Areas with Different Degrees of Restoration

**Figure 5:**
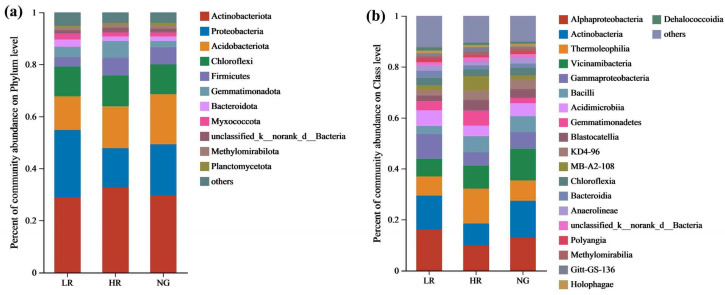
Relative abundance of soil bacterial communities across both phylum and order levels within grasslands of mining sites with different degrees of restoration (%). (**a**) Relative abundance of soil bacterial communities of phylum level; (**b**) Relative abundance of soil bacterial communities of order level.

At the phylum level, the predominant bacterial communities within the soil of grasslands in mining areas with different recovery levels were Actinobacteriota, Proteobacteria, Acidobacteriota, Chloroflexi, and Firmicutes. The proportional representation of bacterial populations varies among grassland ecosystems at varied stages of recovery (Figure 5a). As the recovery levels advance within the grasslands of the mining zones, the relative abundance of Actinobacteriota gradually increases, with relative abundances of 28.91%, 32.69%, and 29.62% in LR, HR, and NG, respectively. The proportional representation of Proteobacteria exhibited a declining pattern initially, then reversed into an ascending trend, with relative abundances of 25.87%, 15.07%, and 19.57% in LR, HR, and NG, respectively. The proportional representation of Acidobacteriota demonstrated a gradual increasing pattern, where the relative abundances were 12.89%, 16.06%, and 19.38% in LR, HR, and NG, respectively. Chloroflexi showed a minor increasing trend, with relative abundances of 11.47%, 11.83%, and 11.50% in LR, HR, and NG, respectively. Firmicutes exhibited a gradually increasing trend, with relative abundances of 3.68%, 6.91%, and 6.55% in LR, HR, and NG, respectively. The proportional representations of other bacterial populations at the phylum level were relatively low, ranging from 0.77% to 2.82%.

At the class level, the predominant bacterial communities in the soils of grasslands in mining areas with different recovery levels were Alphaproteobacteria, Actinobacteria, Thermoleophilia, Vicinamibacteria, Gammaproteobacteria, and Bacilli. The relative abundance of bacterial communities varies among grassland ecosystems across varied recovery stages (Figure 5b). With increasing recovery levels in the mining-area grasslands, the proportional representations of Alphaproteobacteria exhibited a decreasing pattern, where the relative abundances were 16.12%, 9.90%, and 13.03% in LR, HR, and NG, respectively. The proportional representations of Actinobacteria exhibited a downward trend initially, followed by an upward trend, with proportional abundances of 13.30%, 8.53%, and 14.28% in LR, HR, and NG, respectively. Thermoleophilia displayed a rising trend initially, then shifted to a decreasing pattern, with relative abundances of 7.50%, 13.67%, and 8.04% in LR, HR, and NG, respectively. Vicinamibacteria exhibited an increasing trend, with relative abundances of 7.00%, 9.11%, and 12.48% in LR, HR, and NG, respectively. Gammaproteobacteria showed a decreasing trend, with relative abundances of 9.74%, 5.16%, and 6.50% in LR, HR, and NG, respectively. Bacilli exhibited a decreasing trend, with relative abundances of 3.09%, 6.34%, and 6.30% in LR, HR, and NG, respectively. The proportional representations of other bacterial populations at the class level remained relatively modest, spanning from 0.99% to 2.73%.

#### 3.4.3. Examination of the Hierarchical Structure of Soil Bacterial Populations in Grassland Regions within Mining Zones across Varying Levels of Ecological Restoration

**Figure 6:**
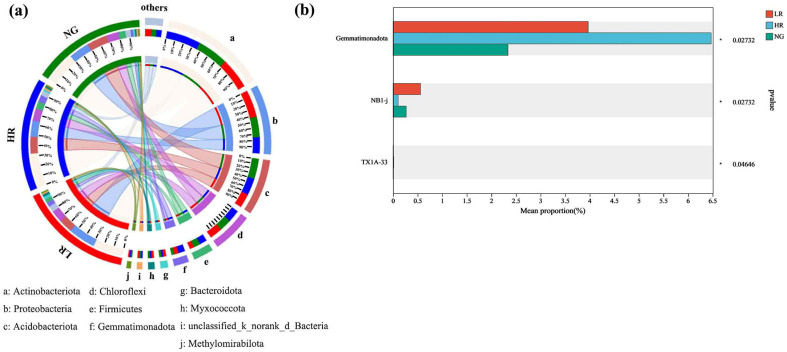
Differences in the gate-level structure of soil bacterial communities in grasslands of mining zones featuring various levels of restoration. (**a**) Discriminant analysis of multilevel species differences in soil bacterial Lefse; (**b**) Differences in soil bacterial species.

In the soil bacterial communities of grasslands in mining areas with different recovery levels, Actinobacteriota was the most abundant bacterial phylum, succeeded by Proteobacteria, Acidobacteriota, Chloroflexi, Firmicutes, Gemmatimonadota, Bacteroidota, and Myxococcota (Figure 6a). The predominant microbial phyla within the soil bacterial populations of grasslands located in mining areas with different recovery levels were Actinobacteriota and Proteobacteria, which were significantly higher than other bacterial phyla. The abundance of Gemmatimonadota within the bacterial populations of soil in grassland areas with HR recovery levels was significantly higher than LR and NG (*p* < 0.05), although no notable difference was observed in the dominant bacterial phyla across grasslands with different recovery levels (Figure 6b).

#### 3.4.4. Variability in the Soil Bacterial Populations within Grasslands of Mining Areas with Different Degrees of Restoration

**Figure 7:**
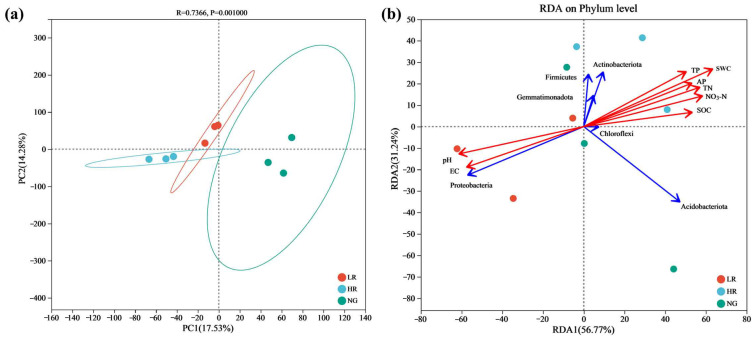
Principal component analysis (PCA) and RDA (redundancy analysis) of soil bacteria in grasslands of mining areas with different degrees of restoration. (**a**) PCA of soil bacteria; (**b**) RDA of soil bacteria.

Principal component analysis (PCA) of soil bacterial populations in grasslands across different stages of recovery within the mining zone revealed marked variations in the distribution of soil bacterial community compositions correlating with the progression of recovery levels. In particular, compared to LR, the soil bacterial communities of HR and NG were slightly closer and less dispersed, with similar compositions between them (Figure 7a). RDA analysis pertaining to soil bacterial populations within grasslands across various recovery stages within the Qianmalong mining area reveals that the cumulative explanatory rates of the first and second sorting axes were 56.77% and 31.24%, respectively, with a total explanatory rate of 88.01%. Actinobacteriota, Firmicutes, Gemmatimonadota, Chloroflexi, and Acidobacteriota demonstrated significant positive associations with AP, TP, TN, NO_3_-N, SOC, and SWC (*p* < 0.05) and significant negative associations with pH levels and EC (*p* < 0.05). Proteobacteria exhibited significant negative correlations with AP, TP, TN, NO_3_-N, SOC, and SWC (*p* < 0.05) and significant positive associations with both pH and EC (*p* < 0.05) (Figure 7b).

#### 3.4.5. Association between Soil Bacterial Communities and Soil Properties in Grasslands of Mining Areas with Different Degrees of Restoration

**Figure 8:**
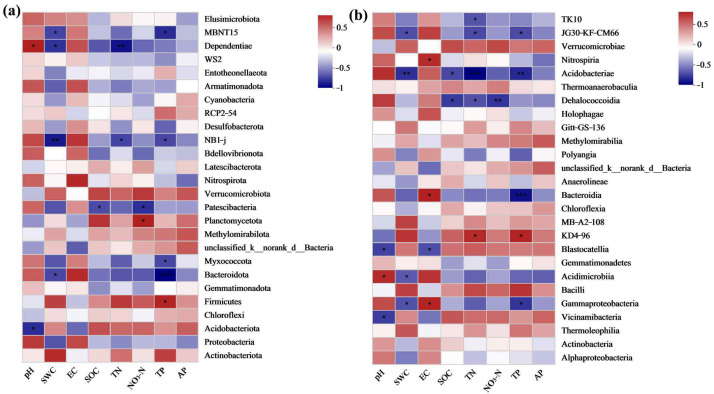
Correlation between major bacteria and soil properties at soil gate and phylum levels in grassland soils of mining sites with different degrees of restoration. (**a**) Correlation between major bacteria and soil properties at soil gate level; (**b**) Correlation between major bacteria and soil properties at soil phylum level. * indicates extremely significant correlation (*p* < 0.05), ** indicates extremely significant correlation (*p* < 0.01), *** indicates extremely significant correlation (*p* < 0.001).

Analysis of the correlation between soil bacteria and soil physicochemical properties in grasslands with different recovery levels in the Qianmalong mining area revealed significant relationships at the phylum and class levels. At the phylum level, Actinobacteriota demonstrated a positive association with soil SWC and TP, while Proteobacteria exhibited a negative correlation with soil SWC. Acidobacteriota showed a significantly negative correlation with soil pH (*p* < 0.05), Firmicutes showed a significant positive relationship with soil TP (*p* < 0.05), and Bacteroidota exhibited a significant negative association with soil SWC (*p* < 0.05) (Figure 8a). At the class level, Acidobacteriae has displayed a significant inverse correlation with soil SWC, TN and TP (*p* < 0.01), whereas Acidimicrobiia demonstrated a significant positive association (*p* < 0.05) and exhibited a significant negative relationship with SWC (*p* < 0.05) (Figure 8b).

#### 3.4.6. Analysis of Soil Bacterial Covariance Networks in Grasslands of Mining Sites with Different Levels of Restoration

**Figure 9:**
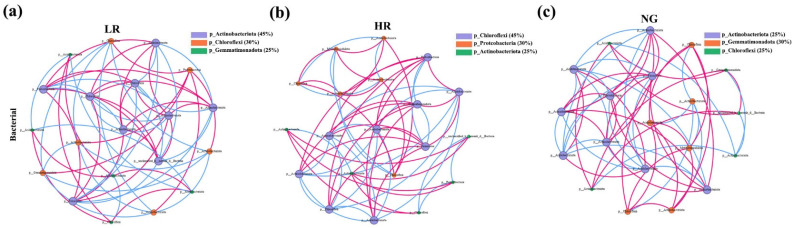
Co-linearity network analysis of the top 20 soil bacteria species in grasslands with different recovery levels in the Qianmalong mining area. (**a**) Co-linearity network analysis of the top 20 soil bacteria species in LR; (**b**) Co-linearity network analysis of the top 20 soil bacteria species in HR; (**c**) Co-linearity network analysis of the top 20 soil bacteria species in NG. Each species is represented by a node of a different color, where node size is inversely related to its degree. Nodes representing species within the same phylum share identical colors. Positive correlations are denoted by red lines, negative correlations by blue lines, and edges signify significant Spearman correlations with a *p*-value less than 0.05 (*p* < 0.05), the same as below.

Soil bacteria co-linearity network analysis can be used to reflect the differences in bacterial interactions in grasslands with different recovery levels in the Qianmalong mining area. By calculating the correlation between species, a species correlation network diagram was constructed, mainly reflecting the complexity of the bacterial co-linearity network through measures such as the distribution of node degrees, the diameter of the network, the network’s average shortest path length, node connectivity, and measures of the closeness centrality, betweenness centrality, and the network’s transitivity. The soil bacteria networks of HR and NG had higher network edges than LR, with NG showing the highest network diameter, density, node distribution of degrees, mean shortest path length, mean clustering coefficient, as well as average degrees. SG soil bacteria had a higher average path lengths but lower complexity. The complexities of the HR and NG networks were higher, and the HR soil bacteria network was closer to the NG soil bacteria network, indicating that HR has the best recovery level (Figure 9).

### 3.5. Impact of Vegetation Recovery on Soil Fungal Populations in High-Altitude Mining Regions

#### 3.5.1. Variation in Soil Fungal Community Composition across Grasslands in Mining Areas with Varying Levels of Restoration

**Figure 10:**
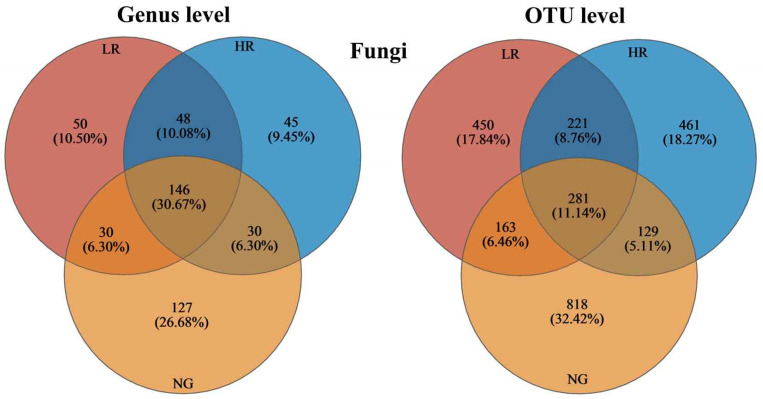
Numbers and proportions of common and unique genera and OTUs of soil fungal communities in grasslands in mining regions exhibiting varying levels of restoration.

In the soils of grasslands with different recovery levels in the Qianmalong mining area, there were 146 genera in common, accounting for 30.67% of all identified genera, and 281 common OTUs, accounting for 11.14% of the entirety of OTUs (Figure 10). Specifically, the count of OTUs in the fungi of HR soils exceeded that in LR soils (461 > 450), and the number of genera in NG soils exceeded that in HR soils (127 > 45). Additionally, the number of OTUs in the fungi of NG soils exceeded that in HR soils (818 > 461).

#### 3.5.2. Relative Abundance of Soil Fungal Community Composition in Grasslands of Mining Areas with Different Degrees of Restoration

**Figure 11:**
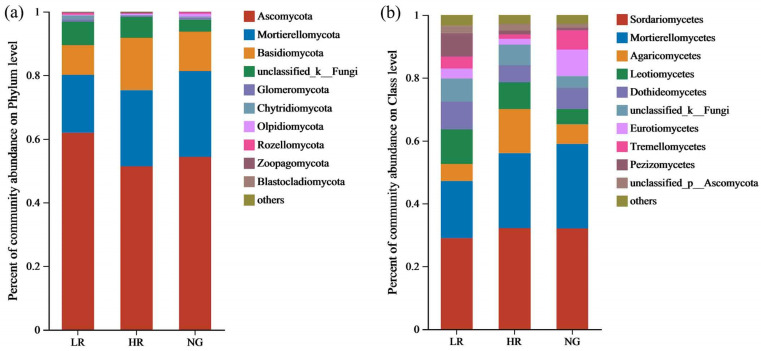
Proportional presence of soil fungal populations at the phylum and order levels in grasslands of mining sites with different degrees of restoration (%). (**a**) Relative abundance of soil fungal communities of phylum level; (**b**) Relative abundance of soil fungal communities of order level.

At the phylum level, the predominant fungal populations in the soils of grasslands with different recovery levels in the Qianmalong mining area primarily comprised Ascomycota, Mortierellomycota, Basidiomycota, unclassified_k_Fungi, and Glomeromycota. The relative abundance of fungal communities varied across different recovery levels (Figure 11a). With the increase in grassland recovery level, the proportional representation of Ascomycota gradually increased, with values of 61.94%, 51.38%, and 54.34% in LR, HR, and NG, respectively. Mortierellomycota showed an increasing trend in relative abundance, with values of 18.19%, 23.90%, and 26.97% in LR, HR, and NG, respectively. Basidiomycota exhibited an increasing trend in relative abundance, with values of 9.36%, 16.52%, and 12.42% in LR, HR, and NG, respectively. The proportional representation of unclassified_k_Fungi showed a decreasing trend, with values of 7.36%, 6.53%, and 3.73% in LR, HR, and NG, respectively. Glomeromycota displayed a downward trend in relative abundance, featuring values of 0.81%, 0.30%, and 0.67% in LR, HR, and NG, respectively. The relative abundance of other phylum-level fungal communities was relatively low, ranging from 0.02% to 0.55%.

At the class level, the predominant fungal communities in the soils of grasslands with different recovery levels in the Qianmalong mining area were Sordariomycetes, Mortierellomycetes, Agaricomycetes, Leotiomycetes, and Dothideomycetes. The relative abundance of fungal communities varied across different recovery levels (Figure 11b). With the increase in grassland recovery level, the proportional presence of Sordariomycetes showed an increasing trend, with values of 28.96%, 32.12%, and 32.02% in LR, HR, and NG, respectively. Mortierellomycetes exhibited a gradually increasing trend in relative abundance, with values of 18.19%, 23.90%, and 26.96% in LR, HR, and NG, respectively. Agaricomycetes showed an increasing trend in relative abundance, with values of 5.43%, 14.04%, and 6.21% in LR, HR, and NG, respectively. Leotiomycetes demonstrated a decreasing trend in relative abundance, with values of 10.98%, 8.51%, and 4.85% in LR, HR, and NG, respectively. Dothideomycetes exhibited a decreasing trend in relative abundance, with values of 8.89%, 5.48%, and 6.77% in LR, HR, and NG, respectively. The proportion of other class-level fungal populations was relatively low, ranging from 1.22% to 3.85%.

#### 3.5.3. Analysis of the Gate-Level Composition of Soil Fungal Communities within Grasslands in Mining Regions Exhibiting Varying Levels of Restoration

**Figure 12:**
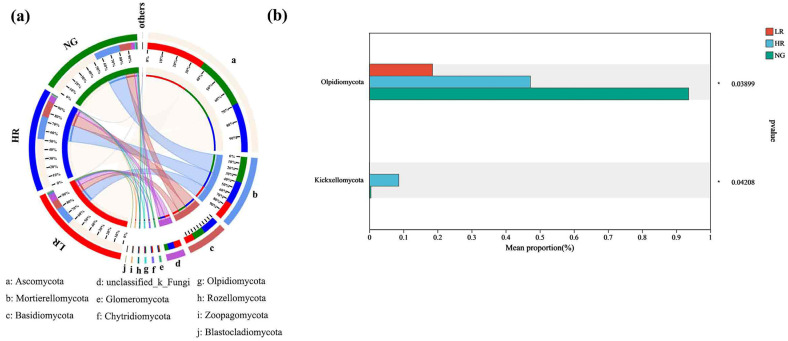
Differences in gate-level makeup of soil fungal populations within grasslands across mining regions at varying restoration levels. (**a**) Discriminant analysis of multilevel species differences in soil fungal Lefse; (**b**) Differences in soil fungal species.

In the soil fungal communities of grasslands with different recovery concentrations in the Qianmalong mining area, Ascomycota was the most abundant fungal phylum, followed by Mortierellomycota, Basidiomycota, unclassified_k_Fungi, Glomeromycota, Chytridiomycota, and Olpidiomycota (Figure 12a). The predominant fungal phyla in the soil fungal communities of grasslands with different recovery levels were Ascomycota and Mortierellomycota, whose abundances were significantly higher than other fungal phyla. The quantity of Olpidiomycota in the soil fungal communities of grasslands at the HR recovery level was markedly higher than in LR (*p* < 0.05), although no significant discrepancy was observed in the predominant fungal phyla between grasslands of different recovery levels (Figure 12b).

#### 3.5.4. Diversity of Soil Fungal Communities in Grasslands of Mining Regions Exhibiting Varying Levels of Restoration

**Figure 13:**
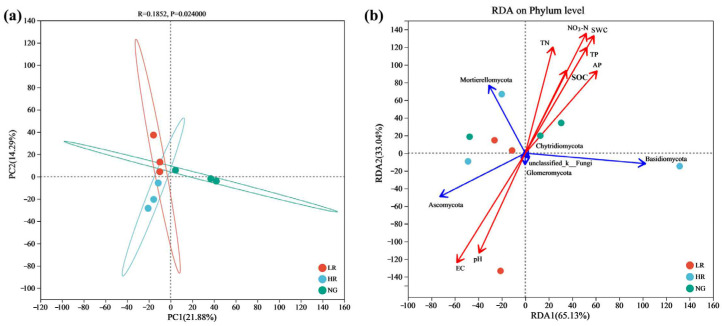
Principal component analysis (PCA) and RDA (redundancy analysis) of soil fungi in grasslands across mining regions at varying restoration stages. (**a**) PCA of soil fungi; (**b**) RDA of soil fungi.

The PCA analysis of soil fungal communities in the grasslands across various restoration stage levels in the Qianmalong mining area revealed significant differences in community structure as restoration levels progressed. In contrast to the NG, the fungal communities in LR and HR grasslands exhibited closer distances and smaller dispersions, indicating a higher similarity between LR and HR communities (Figure 13a). RDA analysis further showed that in the soil–fungal population relationship within the rehabilitated grasslands of the Qianmalong mining region, the primary and secondary ordination axes accounted for 65.13% and 33.04% of the variation, respectively, with a cumulative explanatory rate of 98.17%. Mortierellomycota, Chytridiomycota, Glomeromycota, and unclassified_k_Fungi showed a significant positive association with AP, TP, TN, NO_3_-N, SOC, and SWC (*p* < 0.05), while showing a negative relationship with soil pH and EC. Conversely, Ascomycota showed significant negative correlations with AP, TP, TN, NO_3_-N, SOC, and SWC (*p* < 0.05), yet exhibited significant positive associations with pH and EC (*p* < 0.05) (Figure 13b).

#### 3.5.5. Correlation between Soil Fungal Communities and Soil Properties in Grasslands of Mining Regions Exhibiting Varying Levels of Restoration

**Figure 14:**
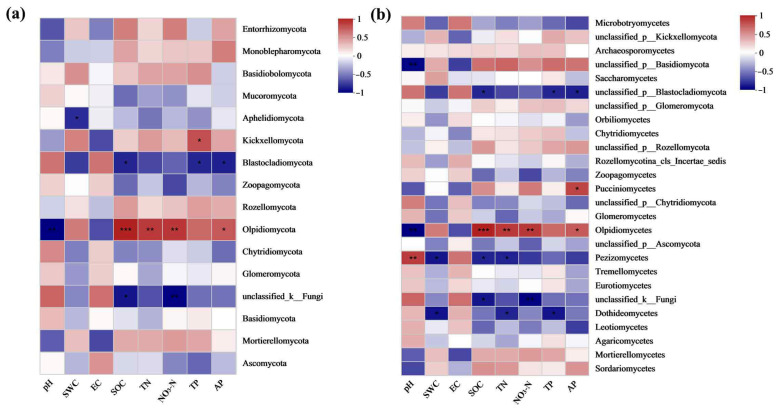
Correlation between major fungi and soil properties at soil gate and phylum levels in grassland soils of mining sites with different degrees of restoration. (**a**) Correlation between major fungi and soil properties at soil gate level; (**b**) Correlation between major fungi and soil properties at soil phylum level. * indicates extremely significant correlation (*p* < 0.05), ** indicates extremely significant correlation (*p* < 0.01), *** indicates extremely significant correlation (*p* < 0.001).

The correlation analysis between soil fungi and soil physicochemical factors in the grasslands across various restoration stages levels in the Qianmalong mining area indicates, at the phylum level, a significant negative correlation between unclassified_k_Fungi and SOC and NO_3_-N (*p* < 0.05), while Olpidiomycota demonstrated a highly significant positive relationship with SOC (*p* < 0.001) and a significant positive correlation with TN and NO_3_-N (*p* < 0.05) (Figure 14a). At the class level, Olpidiomycetes exhibited a significant positive association with TN and NO_3_-N (*p* < 0.05) and a highly marked positive association with SOC (*p* < 0.001) (Figure 14b).

#### 3.5.6. Analysis of Soil Fungal Covariance Networks in Grasslands of Mining Sites with Different Levels of Restoration

**Figure 15:**
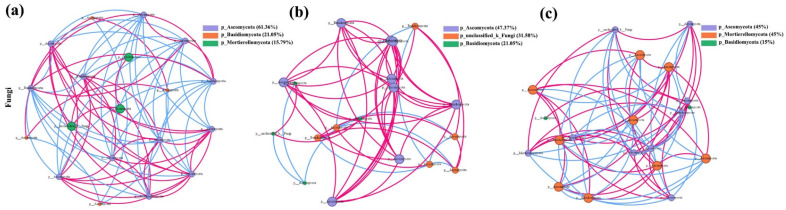
Co-linearity network analysis of the top 20 soil fungi species in grasslands with different recovery levels in the Qianmalong mining area. (**a**) Co-linearity network analysis of the top 20 soil fungi species in LR; (**b**) Co-linearity network analysis of the top 20 soil fungi species in HR; (**c**) Co-linearity network analysis of the top 20 soil fungi species in NG.

The soil fungal networks of HR and NG exhibited higher network edges compared to LR, with NG showing the highest values for network diameter, density, node distribution of degrees, mean shortest path length, and average clustering coefficient, alongside average degree. The HR soil fungal network exhibited a higher average path length but lower complexity, while LR and NG networks displayed higher complexity. The difference between HR and NG soil fungal networks suggests that the restoration of HR soil fungi may require a longer time (Figure 15).

## 4. Discussion

### 4.1. Impacts of Varying Degrees of Vegetation Restoration on Plant Diversity

The vegetation coverage and composition of different species can directly reflect the characteristics of vegetation restoration and growth, as well as the soil nutrient richness for plant growth [25]. The variation in vegetation coverage resulting from the same restoration method is mainly due to the distance from the mining pollution source; that is, vegetation growth is poorer closer to the pollution source and more challenging to restore. Conversely, vegetation further from the pollution source exhibits better growth and is easier to restore, often not requiring secondary restoration [26,27]. In the Qianmalong mining area, primarily undergoing artificial restoration, the degree of restoration varies due to indirect factors such as terrain. Among them, the restoration effect of HR is superior, significantly outperforming LR restoration, which requires secondary replanting and multiple repair efforts.

In the Qianmalong mining area, the vegetation of the different restoration levels mainly consists of *Cyperus rotundus*, *Carex* spp., and *Polygonum viviparum*. The higher vegetation community diversity index in the mining area (HR > NG) indicates that the artificial restoration has been effective, providing a theoretical basis for the rehabilitation of alpine mining-impacted grasslands [28]. The species richness in HR areas reaches a maximum of 2.73, indicating the highest level of restoration. The dominant plant types in the grassland are mainly perennial plants, followed by annual plants. The presence of perennial plants suggests a relatively stable functional composition of the plant community. However, as the restoration period is only five years, the soil fertility may only support the growth of seeds sown and may not have allowed for the establishment of other native annual plants through grazing or wind dispersion. This is consistent with the results reported by Wei et al. [29], where communities with low grassland coverage may see the emergence of weed species.

### 4.2. Impacts of Different Restoration Levels of Vegetation on Soil Properties

Changes in SWC, pH, and EC are essential prerequisites for the development of alpine grassland restoration in high-altitude mining areas. Optimal plant growth requires suitable SWC, pH, and EC levels. The process of grassland restoration is closely linked to changes in SWC and additional variables [30,31,32]. In this study, the HR soil in the Qianmalong mining area exhibited lower EC (173.33 μS/cm) and soil pH (4.54), indicating better restoration effectiveness. This can be attributed to the predominance of herbaceous plants in alpine grasslands, with their roots predominantly found within the 0–20 cm layer of the soil. The microbial activity surrounding the root zone of these plants produces significant amounts of CO_2_ through respiration, leading to a reduction in soil pH in the 0–20 cm layer. This trend is evident, as soil pH increases with depth [33,34,35]. Additionally, during the restoration process of the mining area grasslands, soil EC tends to increase with soil depth initially but decreases as the restoration progresses. A lower soil pH in an environment is conducive to plant growth and results in decreased EC and SWC, ultimately leading to increased plant height and biomass [36,37].

In this study, the LR surface vegetation was sparse, resulting in low ground cover. This leads to long-term wind erosion, resulting in lower AP, TP, TN, NO_3_-N, and SOC. LR sites, being closest to the pollution source, may require secondary or multiple restoration efforts due to their location and grazing activities [38]. The LR areas requiring restoration have been identified in this study, indicating the need for ongoing restoration efforts. In contrast, HR mining site restoration grasslands do not require further restoration. Over time, they can reach the flora and chemical characteristics of the soil in natural grasslands. Although artificial intervention can accelerate the proliferation of vegetation and the improvement of soil properties, it still takes time for the ecosystem to adapt to the alpine grassland environment and become stable. This is consistent in accordance with the research by Hu et al. [39], which suggested that soil restoration is a slow process of increasing the land’s potential value and productivity, as well as a slow process of restoring stability to alpine grassland ecosystems in mining areas.

### 4.3. Analysis of Variations in the Diversity of Soil Microbial Communities

After 5 years of artificial restoration, the community diversity of both bacteria and fungi within the soil exhibited significant differences. This suggests that under the influence of different dominant species, the diversity in the composition of soil microbial populations changes differently. Various plant species can generate different microbial communities, directly impacting the composition and relative abundance of microbial bacteria and fungi. This is consistent with the results showing how plants influence microbial diversity. Soil microbial diversity can be directly regulated by dominant plant species, with vegetation primarily affecting changes in microbial species abundance [40,41].

In this study, at the phylum level, the dominant bacterial communities in the restored grasslands of the Qianmalong high-altitude mining area were Actinobacteriota, Proteobacteria, Acidobacteriota, Chloroflexi, Firmicutes, and Gemmatimonadota. The prevalent fungal communities were Ascomycota, Mortierellomycota, Basidiomycota, unclassified_k_Fungi, and Chytridiomycota. The relative abundance of bacterial communities varied across different restoration levels, consistent with most studies on soil microbial populations in high-altitude grasslands [42,43]. Actinobacteria directly degrade cellulose and chitin, serving as the main source of soil fertility, and their spores can withstand adverse environmental factors [44,45]. Acidobacteria are typically found in nutrient-poor soil environments [46]. Firmicutes usually reflect environmental conditions and can serve as an indirect indicator of grassland restoration levels [47,48]. In the process of grassland rehabilitation within the Qilian Mountains mining area, significant changes were observed in soil bacteria and fungi, with bacterial changes being more pronounced than fungal changes. This indicates that bacteria display greater sensitivity to grassland restoration compared to fungal populations [49,50].

## 5. Conclusions

This study found that during the five-year artificial restoration process in the Qilian Mountains alpine mining area, which involved leveling spoil heaps, removing debris, backfilling mines, and reseeding with grass, the restored grassland vegetation was primarily dominated by *Cyperus rotundus*, *Carex* spp., and *Polygonum viviparum*. The level of grassland restoration increased; the number of plant species, their importance values, and plant community diversity gradually increased; soil pH and EC decreased; and concentrations of AP, TP, TN, NO_3_-N, SOC, and SWC gradually increased. In particular, the soil physicochemical properties of HR and NG tended to converge, indicating the most optimal restoration outcomes. With adequate maintenance and changes in restoration degree over time, the soil properties could approximate those of natural grasslands, with the HR restoration method being the most effective in high-altitude mining areas. Significant differences were observed in the β-diversity of soil bacteria and fungi among different restoration levels, with vegetation restoration significantly impacting soil microbial communities, and changes in soil bacteria being more pronounced than those in soil fungi. The restoration process within mining areas is fluctuating, with the soil environment and soil microbiota not yet stabilized. Therefore, it is recommended to optimize the aboveground vegetation and soil restoration models in the later stages, aiming to enhance plant, soil, and microbial diversity and maintain the health and stability of the mining area’s land.

## Figures and Tables

**Table 1:** Basic information on different levels of restoration of grasslands in alpine mining areas.

Recovered Degree	Geography Coordinate	Altitude/m	Above-Ground Biomass (g m^−2^)	Vegetation Cover/%
Low restoration (LR)	36°91′95″ N 102°66′83″ E	2802	128.93 ± 1.19 b	0.36 ± 0.02 b
High restoration (HR)	36°91′94″ N 102°66′86″ E	2811	420.83 ± 3.29 a	0.72 ± 0.05 a
Natural grassland (NG)	36°91′69″ N 102°66′42″ E	2858	457.47 ± 1.75 a	0.68 ± 0.03 a

Note: Different letters appended to the values among treatments denote significant differences at the 0.05 level.

**Table 2:** Basic characteristics of grassland plant communities at different levels of restoration.

LR		HR		NG	
Species	IV	Species	IV	Species	IV
*Cyperus rotundus*	24.53	*Cyperus rotundus*	29.43	*Cyperus rotundus*	21.18
*Carex* spp.	16.71	*Elymus nutans*	25.86	*Elymus nutans*	15.50
*Elymus nutans*	11.54	*Carex* spp.	10.67	*Carex* spp.	15.02
*Daucus carota*	10.46	*Artemisia smithii Mattf*	8.89	*Potentilla chinensis*	13.78
*Potentilla chinensis*	8.88	*Chenopodium glaucum*	7.46	*Oxytropis ochrocephala*	12.93
*Sonchus oleraceus*	4.69	*Sonchus oleraceus*	6.14	*Chenopodium glaucum*	8.50

Note: IV, importance value; LR, low restoration; HR, high restoration; NG, CK natural grassland.

## Data Availability

Data sharing is not applicable to this article.
